# Chidamide enhances cytotoxicity of doxorubicin by promoting autophagy and apoptosis in breast cancer

**DOI:** 10.1186/s12885-023-10774-w

**Published:** 2023-04-17

**Authors:** Jieqing Li

**Affiliations:** Department of Breast Cancer, Center of Cancer, Guangdong Provincial People’s Hospital (Guangdong Academy of Medical Sciences), Southern Medical University, No.123 Huifu West, Yuexiu District, Guangzhou, 510080 Guangdong China

**Keywords:** Chidamide, Doxorubicin, Autophagy, Apoptosis, Breast cancer

## Abstract

**Background:**

Breast cancer (BC) is a prevalent disease that harms women's health, and in-depth investigations of the pathogenesis, treatment, and prevention of BC are the focus of many research programs. Chidamide (CHI) is a histone deacetylase suppressor that depresses histone deacetylase functions, thereby influencing cell growth through an epigenetic mechanism. However, CHI effects upon BC are largely unknown. Present research aimed to confirm the possibility of using CHI combined with chemotherapy drug doxorubicin (DOX) to prevent chemotherapeutic BC resistance in vivo and in vitro.

**Methods:**

In this study, CCK8 (a plate colony formation assay) was applied to detect cell proliferation. Flow cytometry detection showed the apoptotic cell death of both T47D and MCF-7 cells. Nude mouse xenografts were used to detect tumor growth and pulmonary metastasis. High-throughput sequencing was used to detect expression of different genes.

**Results:**

Our data showed that CHI treatment reduced BC cell proliferation, tumor growth, and cell invasion. CHI treatments stimulated BC cell apoptosis by promoting ULK2-mediated autophagy and increasing MCF-7 cell sensitivity to DOX, resulting in decreased tumor growth.

**Conclusion:**

Collectively, our results illustrated that CHI enhanced DOX cytotoxicity by promoting apoptosis and autophagy in BC cells, which advised that CHI could be a candidate drug for BC patient treatments.

**Supplementary Information:**

The online version contains supplementary material available at 10.1186/s12885-023-10774-w.

## Background

Breast cancer (BC) is a life-threatening malignant cancer commonly diagnosed among females around the globe [1]. Despite developments in diagnosis and therapeutic strategies, the prognosis for BC patients is not promising because of high chemoresistance and metastasis frequency [2–5]. Thus, a better understanding of essential signaling pathways and new therapeutic target discovery are crucial to provide a better prognosis for BC patients.

Chidamide (CHI) was the first subtype-selective histone deacetylase inhibitor (HDACi) that China synthesized to develop independently, and it selectively inhibits HDAC10 in class IIb and HDAC3, HDAC2, and HDAC1 in class I [6–9]. CHI is applied to treat BC due to its comfortable administration, good curative effects, strong targeting, and few adverse reactions. In combined therapy, Jiang et al. [10] found that several oncogenic signaling pathways could be simultaneously targeted, which increases the probability of preventing drug resistance in difficult-to-cure advanced BC.

In the present investigation, we tested CHI efficacy using BC cell lines. CHI has a synergistic sensitization effect with chemotherapy drug doxorubicin (DOX), as it enhances DOX cytotoxicity by promoting autophagy and apoptosis in BC cells. Therefore, we also evaluated the potential of combining CHI with DOX to prevent BC chemotherapeutic resistance. Our results provided the experimental foundation for further clinical applications of CHI.

## Methods

### Ethics statement

Our group obtained BALB/c nude mice (aged 4 weeks with 15 ~ 20 g weight) from Shanghai SLAC Laboratory Animal Co., Ltd., Shanghai, China. Ethics Committee at Guangdong Provincial People's Hospital (KY2020-675–01) approved all procedures, which we carried out following ARRIVE guidelines. Surgical processes were performed with anesthesia to minimize suffering. Our group anesthetized mouse through intraperitoneal injection with 30 mg/kg of sodium pentobarbital.

### Cell culture

Our team purchased human BC cell lines (T47D and MCF-7) from American Type Culture Collection (Manassas, VA, USA), which our team cultivated in Dulbecco’s Modified Eagle’s medium (Gibco, Grant Island, NY, USA). We supplied cell lines with 10% FBS in humidified atmosphere including 5% CO_2_ at 37 °C. Our team purchased CHI from Chipscreen Biosciences (Shenzhen, China). We dissolved CHI in DMSO and treated T47D along with MCF-7 cells via different CHI concentrations. We purchased DOX from Rhawn (Shanghai, China), which was dissolved in DMSO to generate different concentrations.

### Next-generation and strand-specific RNA-Seq library

Our team gained total RNA from MCF-7 cells with or without CHI treatment. After agarose electrophoresis and Nanodrop inspection as well as quantification concerning total RNA samples, oligo (dT) magnetic beads (rRNA removal kit was utilized directly if RNA was prokaryotic or degraded) were employed for mRNA enrichment. All RNA sequencing libraries were prepared applying the kit, and the steps included RNA fragment inversion into the first-strand cDNA with random primers, additional dUTP for the synthesis regarding double-strand cDNA terminal repair, second-strand cDNA, addition of A, Illumina matching connector connections, and PCR amplification to prepare the final library. The library that constructed was inspected utilizing Agilent 2100 system (Santa Clara, CA, USA), quantified employing quantitative PCR, which we sequenced using Illumina NovaSeq 6000 sequencer (San Diego, CA, USA).

### Cell viability analysis

Our lab utilized a cell counting kit (CCK8, Dojindo, Japan) to validate CHI or DOX effects on cell viability separately or integratively. We incubated cells in 10% CCK8 diluted in normal culture medium at 37 °C until visual color transformation happened. Our team measured proliferation rates at 1, 2, and 3 days post treatment. Our lab detected absorbance in each well utilizing microplate reader set at 450 nm.

### Transwell migration assay

We assessed cell migration utilizing Costar Transwell cell culture inserts (Corning Incorporated, Corning, NY, USA) following manufacturer’s guidelines. After 1 d incubation, our team erased cells from transwell chamber upper surfaces using cotton swabs. We fixed these cells on the lower surfaces using methanol for 10 min, which we stained with crystal violet. We photographed and calculated the percentage of stained cells in five fields that were selected at random.

### Plate colony formation assay

We used MCF-7 and T47D cells treated with CHI, DOX, or CHI + DOX at different concentrations to prepare count cell suspensions. We added 200 cells to wells of 6-well plates and further cultured them in an incubator for 2 weeks. We changed the cell culture medium every 3 days and observed the cell state and captured photographs using a fluorescence microscope prior to the experiment ending. The cells were cleaned twice utilizing phosphate buffered saline (PBS). Afterwards, 500 µL crystal violet solution (0.1%) were put to each well to stain the cells for 5 min. After washing the cells three times utilizing ddH_2_O, we photographed the cells under the microscope using a digital camera.

### Annexin V staining

We used the Annexin V-FITC/PI Assay Kit (ImmunoWay, Plano, TX, USA) following manufacturer’s recommendations to assess apoptosis of the cells in each treatment. We washed the cells twice (1 × 10^5^) via PBS and then suspended them in 100 μL of binding buffer, which we stained using 5 μL of Annexin V-FITC for 0.5 h in dark. We added 5 μL of propidium iodide for 5 min and then added binding buffer to bring the total volume to 250–300 μL. We measured fluorescence utilizing flow cytometer (BD, Franklin Lakes, NJ, USA). Quantitative values were calculated as the average Annexin V-positive cell percentiles of three independent experiments.

### In vivo experiments

To establish the nude mice models of BC, 2 × 10^6^ MCF-7 cells without Matrigel were injected into nude mice flank. 14 days after cell injection, we randomly divided tumor-bearing mice into 4 groups with 5 mice per subgroup: i) control group (normal saline); ii) DOX group (5 mg DOX/kg body weight); iii) CHI group (5 mg CHI/kg body weight); and iv) CHI + DOX group (5 mg CHI + 5 mg DOX/kg body weight). We injected the drugs every 3 days to monitor tumor volumes until we euthanized the mouse. We measured tumor weight and volume at the end of the experiment. The tumor volume was computed by 1/2 (length × width × width).

For the tumor metastasis analysis, our team suspended luminescence-labeled MCF-7 cells (2 × 10^5^) in sterile PBS and then injected into individual nude mice tail vein. After 4 weeks, we evaluated lung metastasis by applying an in vivo bioluminescence imaging system. The metastatic foci number in lung tissues was obtained following hematoxylin and eosin (HE) staining.

### Immunohistochemical and immunofluorescence analyses

We fixed tumor tissues in 4% paraformaldehyde solution, which we embedded in paraffin sliced them into 5 μm thick sections. Technician stained them using HE or antibodies against Ki67 and examined them under Zeiss Axioplan 2 microscope (Carl Zeiss AG, Oberkochen, Germany) equipped with digital instrument to confirm cell growth.

For the terminal deoxynucleotidyl transferase dUTP nick end labeling (TUNEL) assay in vivo, we first fixed tumor tissues by applying 4% paraformaldehyde and permeabilized them with 1% Triton X-100. We conducted TUNEL process utilizing an in situ cell apoptosis detection kit (Roche, Shanghai, China). We mounted cells in SlowFade Antifade by DAPI (Solarbio, Beijing, China), which we visualized under the Zeiss Axioplan 2 microscope.

For the autophagy assay in vivo, we first fixed tumor tissues with 4% paraformaldehyde, embedded them in paraffin, sliced them into 5 μm thick sections, stained them with LC3 and ULK2, and viewed them under the Axioplan 2 microscope. ImageJ software was used for fluorescence density analysis.

### Statistical analyses

Data are represented by means ± standard deviation (SD). Our team performed statistics analyses using GraphPad Prism (La Jolla, CA, USA) to identify significant differences between groups. We considered *P*-values ≤ 0.05 to be statistical significance. Our team used two-tailed Student’s t-tests to obtain significant differences between 2 groups. One-way analysis of variance with post hoc Bonferroni tests were utilized to compute significant differences among >  = 3 groups.

## Results

### CHI treatment inhibited BC cell proliferation and tumor growth

**Fig. 1:**
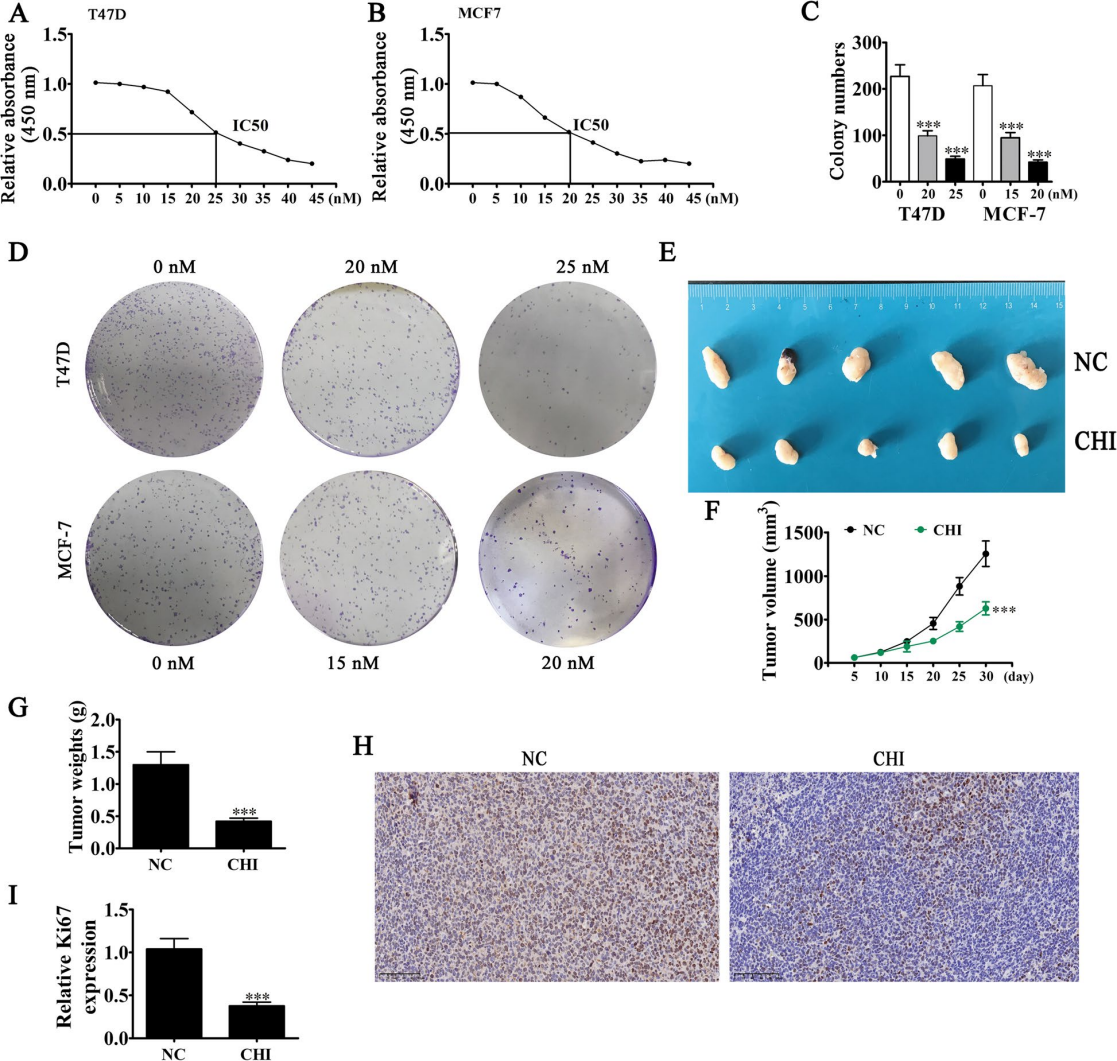
CHI treatment inhibited BC cell proliferation and tumor growth. **A**, **B** CCK8 detection showed the proliferation ability of T47D (A) and MCF-7 (**B**) cells after treatment with different doses of CHI for 24 h. **C**, **D** Colony formation assay results showed T47D and MCF-7 cell proliferation after treatment with different doses of CHI. Data are expressed as means ± SD. ^***^P < 0.001 vs. 0 nM CHI. **E** Images of nude mouse xenografts regarding MCF-7 cells. **F**, **G** Tumor volumes in mice were measured every 5 days. Data are expressed as means ± SD. ^***^P < 0.001 vs. CHI. **H**, **I** Immunohistochemical detection showed the expression of Ki67 in the tumor tissues. Data are expressed as means ± SD. ^***^P < 0.001 vs. 0 nM CHI. Scal bar, 100 μm

The CCK8 detection kit showed that the CHI treatment suppressed BC cell growth depending on concentration in T47D and MCF-7 cells. The half-maximal inhibitory concentrations (IC50s) for MCF-7 and T47D were 20 and 25 nM, respectively (Fig. 1A–B), and the colony formation assay showed that CHI treatment significantly suppressed proliferation of T47D and MCF-7 cells (Fig. 1C–D). The in vivo xenograft mouse assay demonstrated that tumor weight and volume in nude mice injected with MCF-7 cells decreased after CHI treatment (Fig. 1E–G). Additionally, immunohistochemical analysis showed that CHI treatment decreased Ki67 expression in tumor tissues (Fig. 1H–I). These results suggested that CHI treatment suppressed BC proliferation and tumor growth.

### CHI treatment inhibited BC cell invasion

**Fig. 2:**
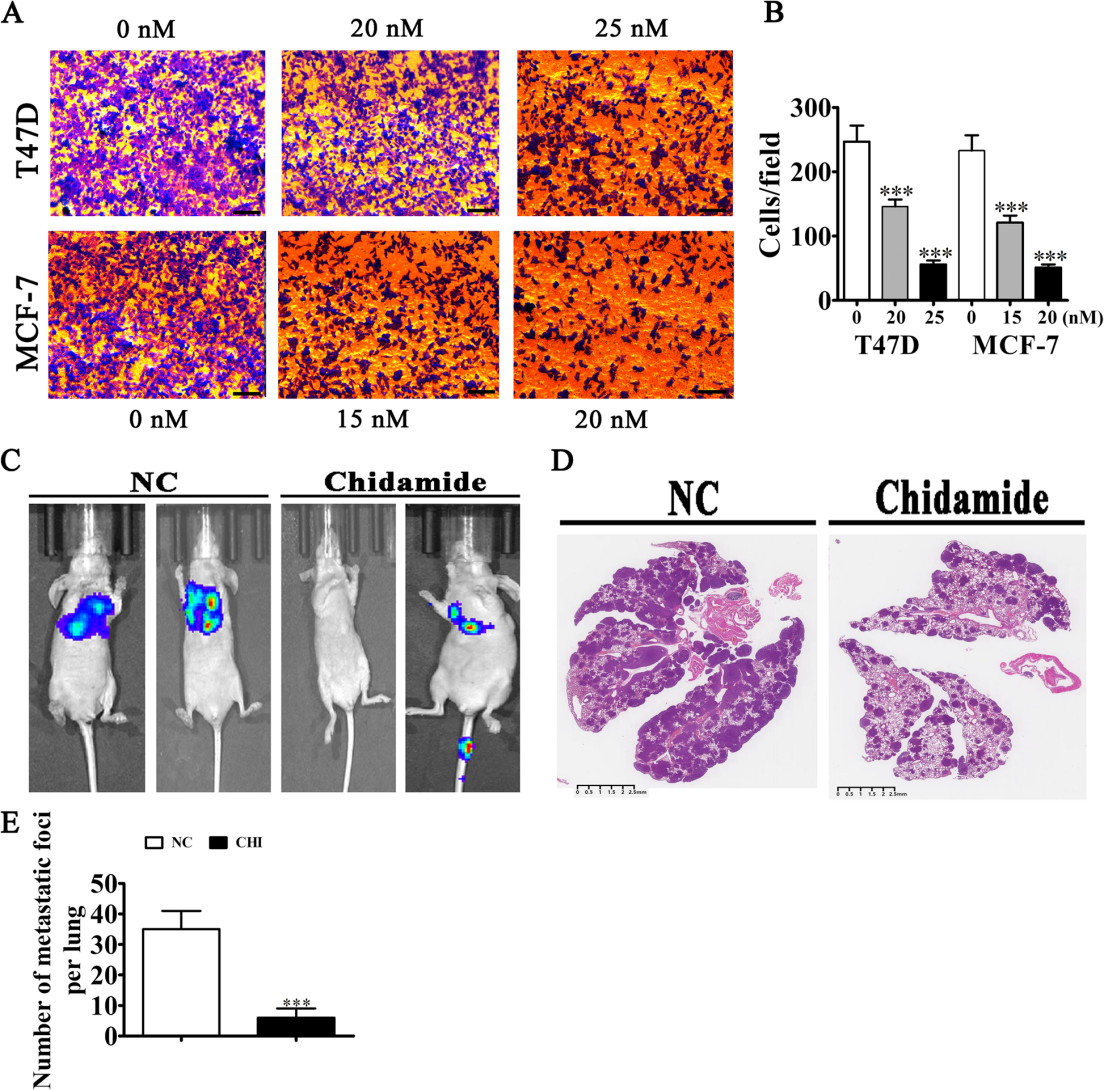
CHI treatment inhibited BC cell invasion. **A**, **B** Transwell data showed the migration ability of both T47D and MCF-7 cells after treatment with CHI. Data are expressed as means ± SD. ^***^P < 0.001 vs. 0 nM CHI. Scal bar, 50 μm. **C** Live imaging detection showed pulmonary metastasis of the MCF-7 cells. **D**, **E** The numbers of metastatic foci in lung tissues were computed following HE staining. Data are expressed as means ± SD. ^***^P < 0.001 vs. NC. Scal bar, 2.5 mm

Results of the transwell migration assay showed that CHI treatment suppressed migration of MCF-7 and T47D cells (Fig. 2A–B). Live imaging detection revealed pulmonary metastasis of MCF-7 cells, and HE staining showed that CHI treatment reduced this metastasis capability by reducing the metastatic foci number in lung tissues (Fig. 2C–E). These findings indicated that CHI treatment inhibited BC cell invasion in vivo and in vitro.

### CHI treatment promoted BC cell apoptosis by promoting autophagy

**Fig. 3:**
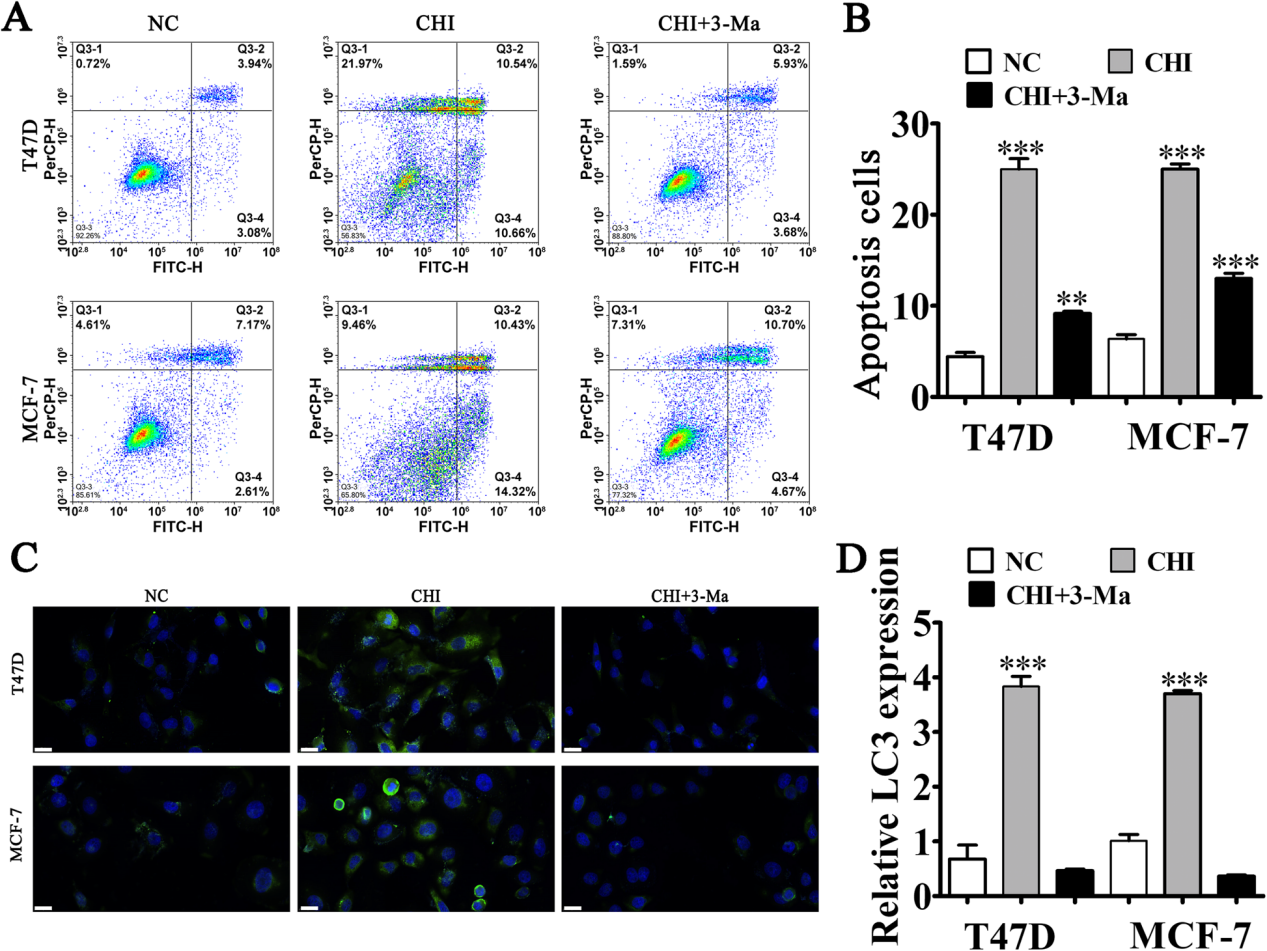
CHI treatment promoted BC apoptosis by promoting autophagy. **A**, **B** Flow cytometry detection showed the apoptosis of both MCF-7 and T47D cells after treatment with CHI and 3-Ma (autophagy inhibitor). Results are expressed as means ± SD. ^**^P < 0.01, ^***^P < 0.001 vs. NC. **C**, **D** Immunofluorescence detection showed the LC3 expression in T47D and MCF-7 cells. The results are expressed as means ± SD. ^***^P < 0.001 vs. NC. Scal bar, 20 μm

Flow cytometry revealed that CHI treatment promoted cell apoptosis in MCF-7 and T47D cells, but 3-Ma (autophagy inhibitor) treatment reversed CHI-induced cell apoptosis (Fig. 3A–B). These results suggested that autophagy played a role in CHI-mediated BC cell apoptosis. Immunofluorescence detection showed that CHI treatment promoted LC3 expression, but 3-Ma treatment inhibited CHI-induced LC3 expression (Fig. 3C–D). Thus, CHI treatment appeared to promote BC cell apoptosis by promoting autophagy.

### CHI treatment increased MCF-7 and T47D cell sensitivity to DOX

**Fig. 4:**
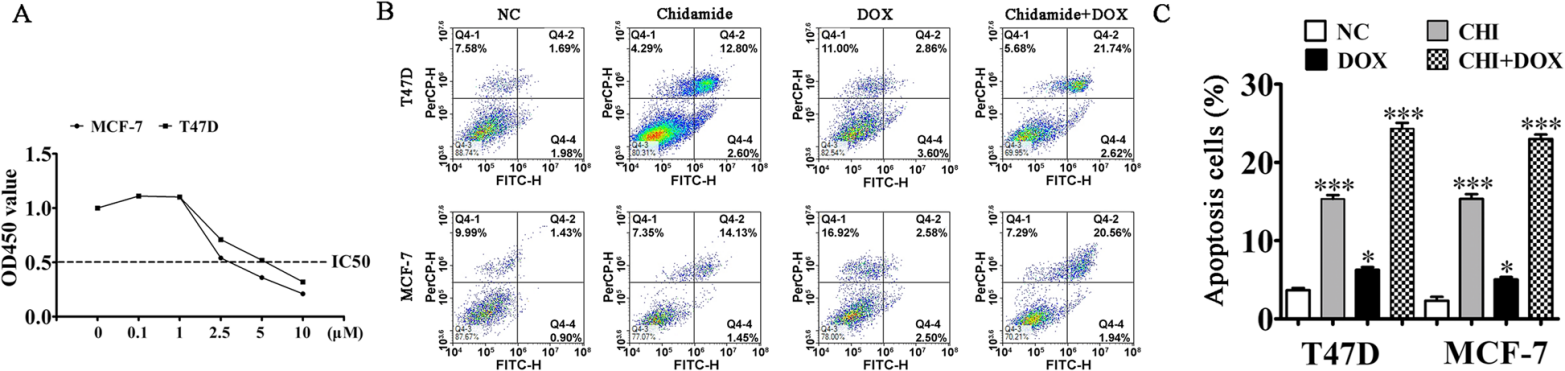
CHI treatment increased the sensitivity of both MCF-7 and T47D cells to DOX. **A** CCK8 detection showed the proliferation ability of T47D and MCF-7 cells after treatment with different doses of DOX for 24 h. **B**, **C** Flow cytometry detection illustrated the MCF-7 and T47D cell apoptosis after treatment with CHI and DOX. The results are expressed as means ± SD. ^*^P < 0.05, ^***^P < 0.001 vs. NC

The CCK8 detection results showed that DOX treatment suppressed cell proliferation in a dose-dependent manner in T47D and MCF-7 cells (Fig. 4A). In CHI + DOX treatment group, flow cytometry revealed that DOX treatment increased CHI-induced apoptosis in T47D and MCF-7 cells (Fig. 4B–C). This result suggested that CHI treatment improved T47D and MCF-7 cell sensitivity to DOX.

### CHI treatment increased MCF-7 cell sensitivity to DOX via increased ULK2-mediated autophagy and decreased tumor growth

**Fig. 5:**
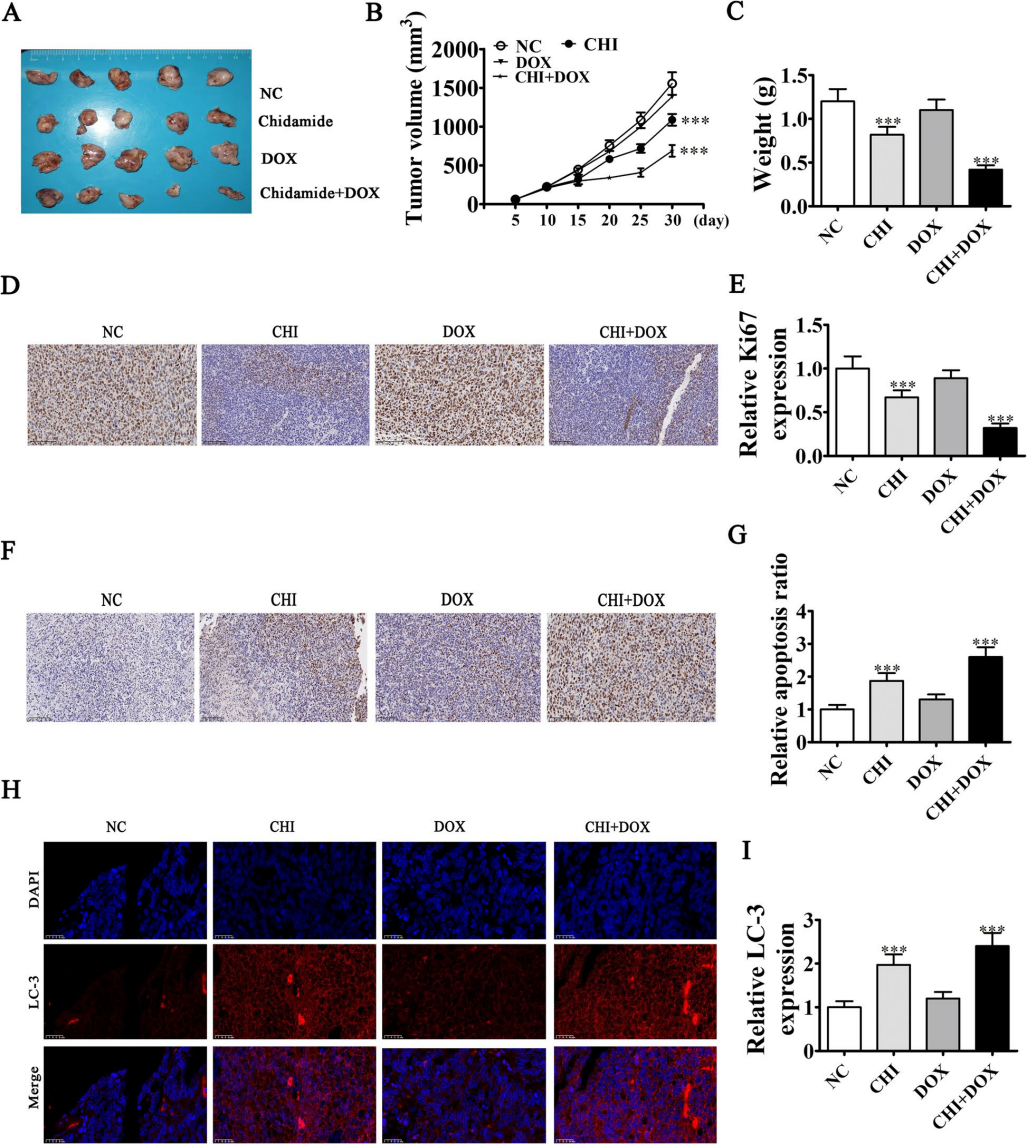
CHI treatment increased the sensitivity of MCF-7 cells to DOX, as indicated by decreased tumor growth. **A** Illustrative images of nude mouse xenografts regarding MCF-7 cells. **B**, **C** Tumor volumes in mice were detected every 5 days. Results are expressed as means ± SD. ^***^P < 0.001 vs. CHI. **D**, **E** Immunohistochemical detection showed the expression of Ki67 in the tumor tissues. Results are expressed as means ± SD. ^***^P < 0.001 vs. NC. Scal bar, 100 μm. **F**, **G** TUNEL staining showed the apoptosis in tumor tissues. Data are expressed as means ± SD. ^***^P < 0.001 vs. NC. Scal bar, 100 μm. **H**, **I** Immunofluorescence detection showed the expression of autophagy related protein LC3 in the tumor tissues. Results are expressed as means ± SD. ^***^P < 0.001 vs. NC. Scal bar, 20 μm

The in vivo xenograft mouse assay showcased that tumor volume and weight in nude mice injected with MCF-7 cells decreased after CHI treatment (Fig. 5A–C) but that treatment with 15 mg/kg of DOX did not inhibit MCF-7 tumor growth. However, treatment with the combination of CHI + DOX significantly decreased MCF-7 tumor growth. Immunohistochemical staining for Ki67 expression also showed that CHI treatment increased the MCF-7 cell sensitivity to DOX through decreased tumor growth (Fig. 5D–E). TUNEL staining showed that the combination of CHI + DOX resulted in significantly higher apoptosis in tumor tissues compared to CHI alone (Fig. 5F–G). Immunofluorescence detection showed that CHI treatment promoted LC3 expression, and autophagy-related protein LC3 expression was also increased with CHI + DOX treatment (Fig. 5H–I). Thus, CHI treatment appeared to have improved MCF-7 cell sensitivity to DOX by promoting autophagy.

**Fig. 6:**
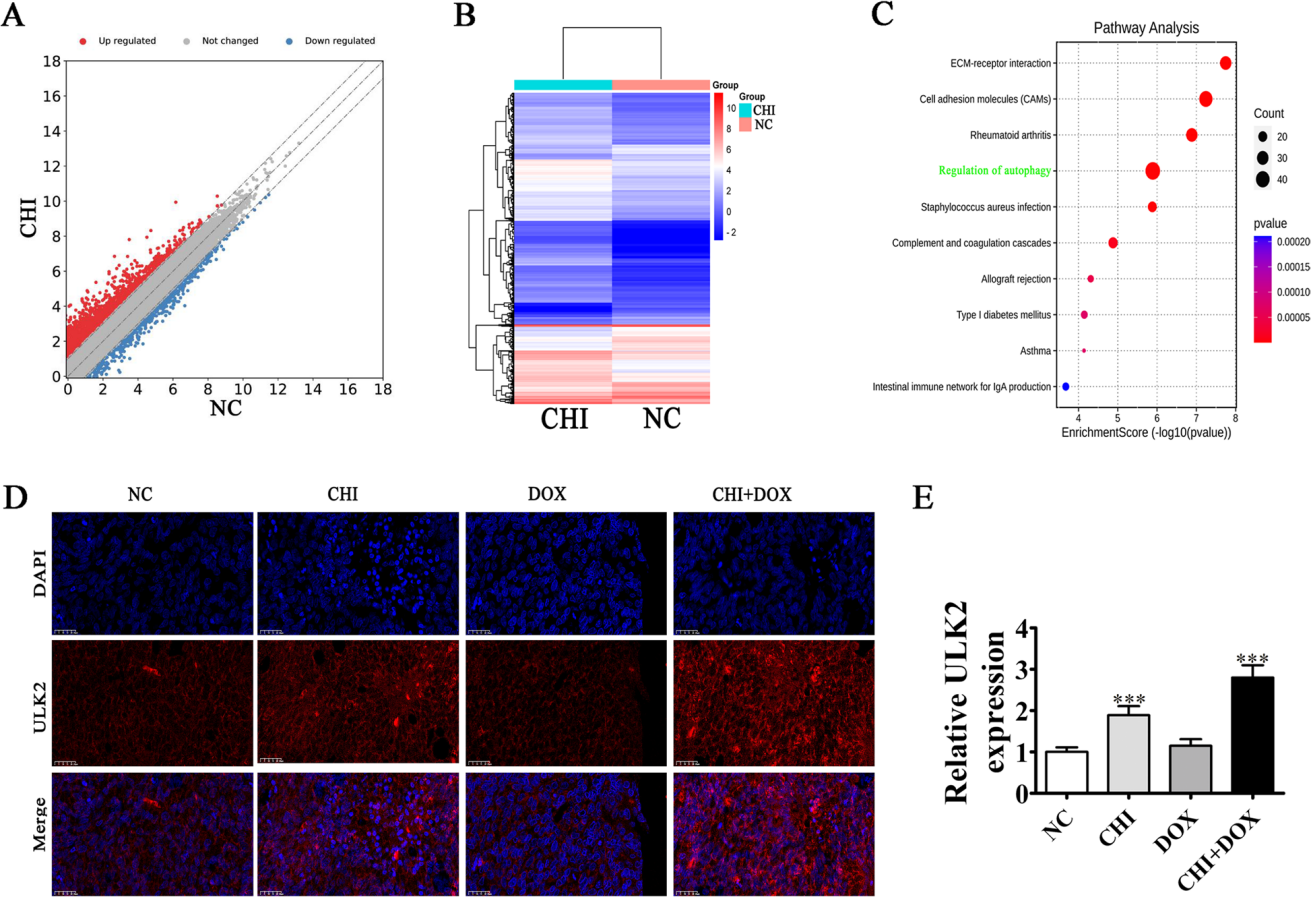
CHI treatment increased ULK2-mediated autophagy. **A** Representative images of the scatter plot. **B** Clustered heat map for MCF-7 cells after CHI treatment compared with the normal control cells (NC). **C** Results of high-throughput sequencing showing differential expression of genes and results of KEGG pathway enrichment analyses after CHI treatment [11]. **D**, **E** Immunofluorescence detection showed ULK2 expression in the tumor tissues. Results are expressed as means ± SD. ^***^P < 0.001 vs. NC. Scal bar, 20 μm

High-throughput sequencing revealed that CHI treatment regarding MCF-7 cells affected gene expression at mRNA level (Fig. 6A and B, Supplementary materials 1). The KEGG pathway enrichment analyses inferred that autophagy pathways participated in CHI-induced autophagy apoptosis via increased ULK2 expression [11] (Fig. 6C, Supplementary materials 2). Immunofluorescence detection showed that CHI treatment promoted ULK2 expression and that autophagy-related ULK2 protein expression was also increased with CHI + DOX treatment (Fig. 6D and E).

## Discussion

DOX is an indispensable chemotherapy for BC treatment. However, tumor cells can become resistant to chemotherapy over time, which is the main reason for chemotherapy failure along with tumor recurrence [12–14]. Drug resistance is a complicated process that involves large-scale mechanisms [15]. Current study found that CHI treatment significantly decreased BC cell proliferation in dose-dependent manner. Additionally, IC50 values of CHI for T47D and MCF-7 cells were 25 and 20 nM, respectively, which were similar to those previously reported [7]. DOX based combinations with TOR, CQ, or CNC were found to affect positively DOX effectiveness and reduce DOX doses applied to BC cells via autophagy modulation [16–18]. Thus, combined treatment might be a therapeutic agent candidate targeting various targets such like autophagy, apoptosis, and notch signaling pathways [19]. We also found that CHI treatment inhibited MCF-7-mediated tumor growth and BC cell invasion in in vivo and in vitro experiments, likely by promoting BC cell apoptosis and autophagy. Treatment with the autophagy inhibitor 3-Ma reversed the CHI-induced BC cell apoptosis, suggesting that CHI triggered autophagy and promoted cell autophagy and apoptosis.

To determine whether CHI could reverse the resistance of BC to DOX, we first determined the IC50 of DOX using CCK8 analysis and found that DOX treatment suppressed cell proliferation. The dose depends on T47D and MCF-7 cells. Subsequent flow cytometry analysis showed that CHI treatment increased T47D and MCF-7 cell sensitivity to DOX. Our in vivo experiment showed that CHI treatment improved MCF-7 cell sensitivity to DOX via decreasing cancer cell growth and increasing autophagy and apoptosis. Previous studies also reported that CHI treatment increased cleaved caspase-7 and LC3 II/I expression [20]. The autophagy assay and Annexin V data showcased that autophagosome microtubule associated protein LC3-II abnormally incremented. Previously, Zhou et al. [21] reported that CHI induced cells to enter the late phase of apoptosis. However, the regulatory mechanisms involved in these processes are still largely unclear.

Therefore, in this study we also used high-throughput sequencing to analyze the abnormal expression of genes at the mRNA level in MCF-7 cells with or without CHI treatment. The result showed that CHI treatment promoted ULK2 expression. ULK2 is homologous to mammalian autophagy-associated proteins that are essential for autophagy initiation [18, 19]. However, validating the underlying intercellular molecular mechanisms and the involvement of ULK2 in autophagy induced by CHI is still needed.

## Conclusion

In conclusion, CHI combined with DOX inhibited cell growth synergistically, increased autophagy activation, and induced apoptosis. This could be an essential mechanism by which CHI combined with DOX can prevent BC drug resistance. More studies are needed to verify the CHI + DOX treatment efficiency and safety in vivo and in vitro to suppress drug resistance in BC.

## Supplementary Information

**Additional file 1.**

**Additional file 2.**

## Data Availability

The data used and/or analyzed during present investigation are available from corresponding author upon requests.

## Abbreviations

BC: Breast cancer
CHI: Chidamide
DOX: Doxorubicin
HDACi: Histone deacetylase inhibitor
PBS: Phosphate buffered saline
HE: Hematoxylin and eosin
